# SCDb: an integrated database of stomach cancer

**DOI:** 10.1186/s12885-020-06869-3

**Published:** 2020-06-02

**Authors:** Erli Gu, Wei Song, Ajing Liu, Hong Wang

**Affiliations:** 1 Department of Gastroenterology, Jing’An District Centre Hospital of Shanghai (Huashan Hospital Fudan University Jing’An Branch), Shanghai, 200040 People’s Republic of China; 2 Yuanzi (Shanghai) Information Technology Co., Ltd, No. 259 Xikang Road, Jing’An District, Shanghai, 200040 People’s Republic of China

**Keywords:** Stomach cancer, Database, Differentially expressed gene, Single nucleotide polymorphism, microRNA

## Abstract

**Background:**

Stomach cancer (SC) is a type of cancer, which is derived from the stomach mucous membrane. As there are non-specific symptoms or no noticeable symptoms observed at the early stage, newly diagnosed SC cases usually reach an advanced stage and are thus difficult to cure. Therefore, in this study, we aimed to develop an integrated database of SC.

**Methods:**

SC-related genes were identified through literature mining and by analyzing the publicly available microarray datasets. Using the RNA-seq, miRNA-seq and clinical data downloaded from The Cancer Genome Atlas (TCGA), the Kaplan-Meier (KM) survival curves for all the SC-related genes were generated and analyzed. The miRNAs (miRanda, miRTarget2, PicTar, PITA and TargetScan databases), SC-related miRNAs (HMDD and miR2Disease databases), single nucleotide polymorphisms (SNPs, dbSNP database), and SC-related SNPs (ClinVar database) were also retrieved from the indicated databases. Moreover, gene_disease (OMIM and GAD databases), copy number variation (CNV, DGV database), methylation (PubMeth database), drug (WebGestalt database), and transcription factor (TF, TRANSFAC database) analyses were performed for the differentially expressed genes (DEGs).

**Results:**

In total, 9990 SC-related genes (including 8347 up-regulated genes and 1643 down-regulated genes) were identified, among which, 65 genes were further confirmed as SC-related genes by performing enrichment analysis. Besides this, 457 miRNAs, 20 SC-related miRNAs, 1570 SNPs, 108 SC-related SNPs, 419 TFs, 44,605 CNVs, 3404 drug-associated genes, 63 genes with methylation, and KM survival curves of 20,264 genes were obtained. By integrating these datasets, an integrated database of stomach cancer, designated as SCDb, (available at http://www.stomachcancerdb.org/) was established.

**Conclusions:**

As a comprehensive resource for human SC, SCDb database will be very useful for performing SC-related research in future, and will thus promote the understanding of the pathogenesis of SC.

## Key points

1. An integrated SC database, SCDb, was constructed.

2. SC-related genes, miRNAs, and SNPs were identified.

3. KM survival curves of 20,264 genes were generated.

4. Gene_disease, CNV, methylation, drug and TF analyses were performed.

5. Convenient links of the String and GENSCAN databases are provided in the SCDb.

## Background

Stomach cancer (SC, also named as gastric cancer) is a type of cancer, which is derived from the stomach mucous membrane [1]. According to the GLOBOCAN 2018 data, SC ranks as the fifth most common neoplasm and the third most leading cause of cancer deaths worldwide, with an estimated count of 783,000 deaths per year [2]. SC is known to reach an advanced stage with relatively poor prognosis due to the non-specific symptoms or no noticeable symptoms appearing in the early stages [3]. The early symptoms of SC include upper abdominal pain, heartburn, loss of appetite and nausea, and the later symptoms include yellowing of the skin and whites of the eyes, weight loss, difficulty in swallowing and excessive vomiting [4]. Besides this, SC also show metastasis from stomach to other tissues or organs, especially the lungs, liver, lining of the abdomen, bones, and lymph nodes [5]. In most of the SC cases (more than 60%), it has been shown to be induced by *Helicobacter pylori* infection [6–8], whereas other causes include smoking, eating pickled vegetables and genetic syndromes [7]. SC is difficult to cure because the patients that are diagnosed with the disease usually have reached an advanced stage [9]. The conventional treatments for SC include surgery [10], radiation therapy, and/or chemotherapy [11].

Although many researchers have performed a series of genomics, proteomics, transcriptomics, and epidemiological studies with regard to SC [12–15], there is only one available database of human gastric cancer, which is the Database of Human Gastric Cancer (DBGC, http://bminfor.tongji.edu.cn/dbgc/index.do) [16]. The DBGC database has integrated human gastric cancer-related biomarkers, drug-sensitive genes, mutations, transcriptomics projects and proteomics projects from different sources, however, some useful information is still excluded from it, as the datasets are greatly dispersive and heterogeneous [16]. Besides this, there is another database Online Mendelian Inheritance in Man (OMIM, http://www.ncbi.nlm.nih.gov/omim) [17], which is an authoritative, comprehensive and timely database that involves the relationship between genotype and phenotype of all human genetic disorders. The miR2Disease [18] and HMDD [19] databases contain comprehensive information about the miRNAs that are related to multiple human diseases. ClinVar database (http://www.ncbi.nlm.nih.gov/clinvar/) provides a repository of relationships among important variants and phenotypes in medical [20]. The above databases majorly focus on molecular mechanisms of various diseases, and not just on SC. Therefore, it is of great importance to develop an integrated SC-specific database which will include gene, gene-disease, miRNA, miRNA_disease, copy number variations (CNVs), single nucleotide polymorphism (SNP), SNP-disease, methylation, drug and transcription factors (TFs).

In this study, we constructed an integrated database of stomach cancer, SCDb, (available at http://www.stomachcancerdb.org/) by retrieving the databases and literature mining and by performing bioinformatics analysis of the publicly available datasets. This human SC database might help researchers to investigate and provide more information about the human SC-related molecules from several clinical aspects.

## Methods

### Data collection

**Fig. 1:**
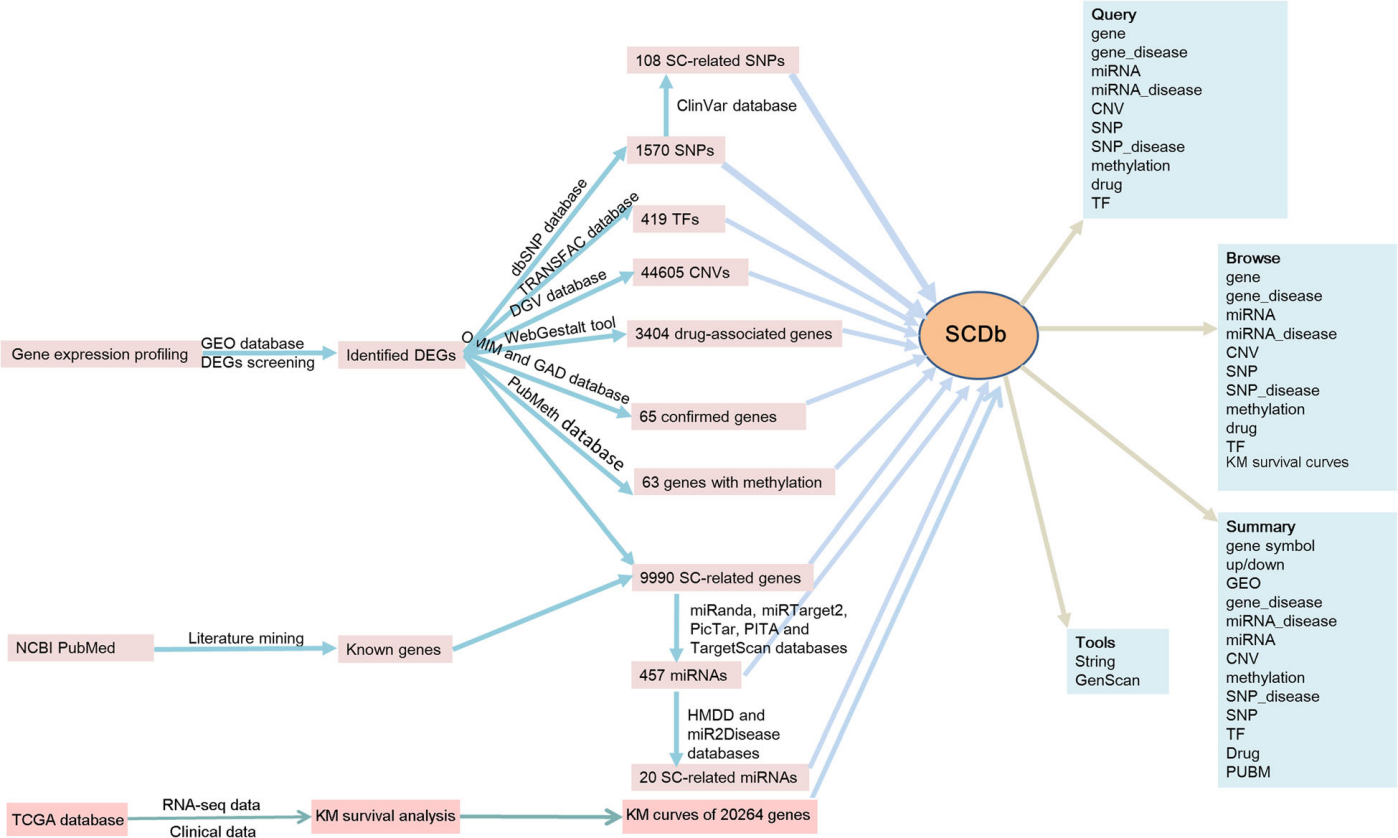
The construction of SCDb. SCDb, Database of Stomach Cancer; NCBI, National Center for Biotechnology Information; GEO, Gene Expression Omnibus; DEGs, differently expressed genes; SC, stomach cancer; SNPs, single nucleotide polymorphisms; TFs, transcription factors; CNVs, copy number variations; miRNAs, microRNAs; KM: Kaplan-Meier

Relevant datasets were retrieved from the National Center for Biotechnology Information (NCBI) database, Gene Expression Omnibus (GEO, http://www.ncbi.nlm.nih.gov/geo/) database, The Cancer Genome Atlas (TCGA, https://cancergenome.nih.gov/) database [21], and by mining of literature from the PubMed database. Subsequently, the selected datasets were processed in accordance with the procedure presented in Fig. 1.

The microarray datasets correlated to SC were selected for further analyses based on the following criteria: (1) the corresponding samples should include both, tumor and normal samples; (2) the corresponding subjects were humans. In contrast, microarray datasets related to gene knockout, drug screening, and time series analysis were excluded. In total, 6 microarray datasets were selected, including GSE13195, GSE19826, GSE2685, GSE27342, GSE33651 and GSE56807 (updated by May, 1, 2014), which were based on GPL5175 [HuEx-1_0-st] Affymetrix Human Exon 1.0 ST Array [transcript (gene) version] and GPL5188 [HuEx-1_0-st] Affymetrix Human Exon 1.0 ST Array [probe set (exon) version], GPL570 [HG-U133_Plus_2] Affymetrix Human Genome U133 Plus 2.0 Array, GPL80 [Hu6800] Affymetrix Human Full Length HuGeneFL Array, GPL5175 [HuEx-1_0-st] Affymetrix Human Exon 1.0 ST Array [transcript (gene) version], GPL2895 GE Healthcare/Amersham Biosciences CodeLink Human Whole Genome Bioarray, and GPL5175 [HuEx-1_0-st] Affymetrix Human Exon 1.0 ST Array [transcript (gene) version], respectively. The clinical information of the samples used in different microarray datasets is listed in Supplemental Table 1.

The RNA-seq and miRNA-seq datasets for level 3 analysis were downloaded from TCGA (version 2016_01_28) database [21], including the expression data of 20,264 genes and clinical data of 411 SC patients.

Mining the literature from PubMed database was mainly based on previously known SC-related genes, the corresponding up−/down-regulation information, and the corresponding sentences. The key words used for identification of previously known SC-related genes are as follow: gastric carcinoma; gastric cancer; stomach cancer; cancer of the stomach; and carcinoma of stomach. The deadline of data retrieval was Jun 30, 2014.

### Identification of SC-related genes

After microarray datasets were downloaded and selected, the raw microarray data were pre-processed according to the corresponding annotation information in different platforms. For multiple probes mapping to one gene, their average value was calculated and was considered as the final gene expression value. Afterwards, the differentially expressed genes (DEGs) between the SC and normal samples were identified using the limma package [22] in R suite. The genes with *p* < 0.05 and |log_2_ fold change (FC)| > 1 were used as the cut-off for identifying DEGs. For subsequent analysis, the identified DEGs and the previously known SC-related genes obtained by mining the related literature from PubMed database were merged as SC-related genes.

### MiRNAs and SC-related miRNAs

The miRNAs targeting the SC-related genes were identified using miRanda (release: August 2010) [23], miRTarget2 (version 4) [24], PicTar (release: March 2007) [25], PITA (release: August 2008) [26], and TargetScan (version 6.2) [27] databases. miRNA targets that were predicted by no less than 3 databases were used as the threshold. Using a combined search with the HMDD (updated on Sep, 9, 2012) [19] and miR2Disease (updated on Apr, 14, 2011) [18] databases, the previously known SC-related miRNAs targeting SC-related genes were identified.

### Analysis of the survival curve of genes

According to the analysis of RNA-seq and miRNA-seq datasets in level 3downloaded from TCGA, the SC patients were divided into low expression and high expression groups based on the median expression value. Combined with their clinical data, the Kaplan-Meier (KM) survival curves of overall survival (OS) between the above indicated two groups were generated using the survival package [1] in R, and the significant difference between the two groups were determined using the log-rank test.

### SNPs and SC-related SNPs

The SC-related somatic mutations data in level 2 were downloaded from TCGA database. Then, the SNPs-related to the identified DEGs were extracted and annotated according to the Single Nucleotide Polymorphism database (dbSNP, http://www.ncbi.nlm.nih.gov/SNP, updated on May, 29, 2014) [28]. Moreover, SC-related SNPs were selected using the ClinVar database [20].

### TF, CNV, drug, disease and methylation analyses

The TFs targeting the identified DEGs were predicted using the TRANSFAC database [29]. The CNVs in the identified DEGs were predicted using the Database of Genomic Variants (DGV, http://projects.tcag.ca/variation/) [30]. Meanwhile, the drug analysis was carried out using the WebGestalt (version 2, http:// bioinfo.vanderbilt.edu/webgestalt/) online tool [31], with *p* < 0.01 and gene number ≥ 10 as the thresholds. Using DAVID software [32], the enrichment analysis of the identified DEGs was performed based on the OMIM database [17] and the genetic association database (GAD, http://geneticassociationdb.nih.gov) [33], with p < 0.05 and gene number ≥ 2 as the cut-off criteria. In addition, methylation analysis of the identified DEGs was performed using the PubMeth database (http://matrix.ugent.be/pubmeth/) [34].

## Results

### Data collection and analysis

Upon analyzing the microarray datasets and mining the literature, a total of 9990 SC-related genes (including 8347 up-regulated genes and 1643 down-regulated genes) were identified, among which, 65 genes were further confirmed as SC-related genes based on the information available on the GAD and OMIM databases. Based on miRanda, miRTarget2, PicTar, PITA and TargetScan databases, 457 miRNAs targeting the SC-related genes were screened and identified. Combined with HMDD and miR2Disease databases, 20 previously known SC-related miRNAs were found to target these SC-related genes. According to the dbSNP database, 1570 SNPs were annotated in the identified DEGs. Thereafter, 108 SC-related SNPs were further selected using the ClinVar database. Through TF, CNV, drug, and methylation analyses, 419 TFs, 44,605 CNVs, 3404 drug-associated genes, and 63 genes with methylation were identified, respectively. In addition to this, using the RNA-seq and clinical datasets, survival analysis for generating the KM survival curves of 20,264 genes was performed, and a total of 2126 genes were identified, whose expression was significantly correlated with the survival time (days).

### Database construction

The SCDb database (available at http://www.stomachcancerdb.org/) was constructed as an integrated database of SC, which is based on the above mentioned retrieved data. SCDb would provide effective help from the perspective of bioinformatics based studies on gastric cancer.

### Database usage instructions

SCDb provides search engines for Query, Browse, and Summary and tools to perform query search to retrieve detailed information on gene, gene-disease, miRNA, miRNA_disease, CNV, SNP, SNP_disease, methylation, drug and TF, for which gene symbol could serve as the query key word.

**Fig. 2:**
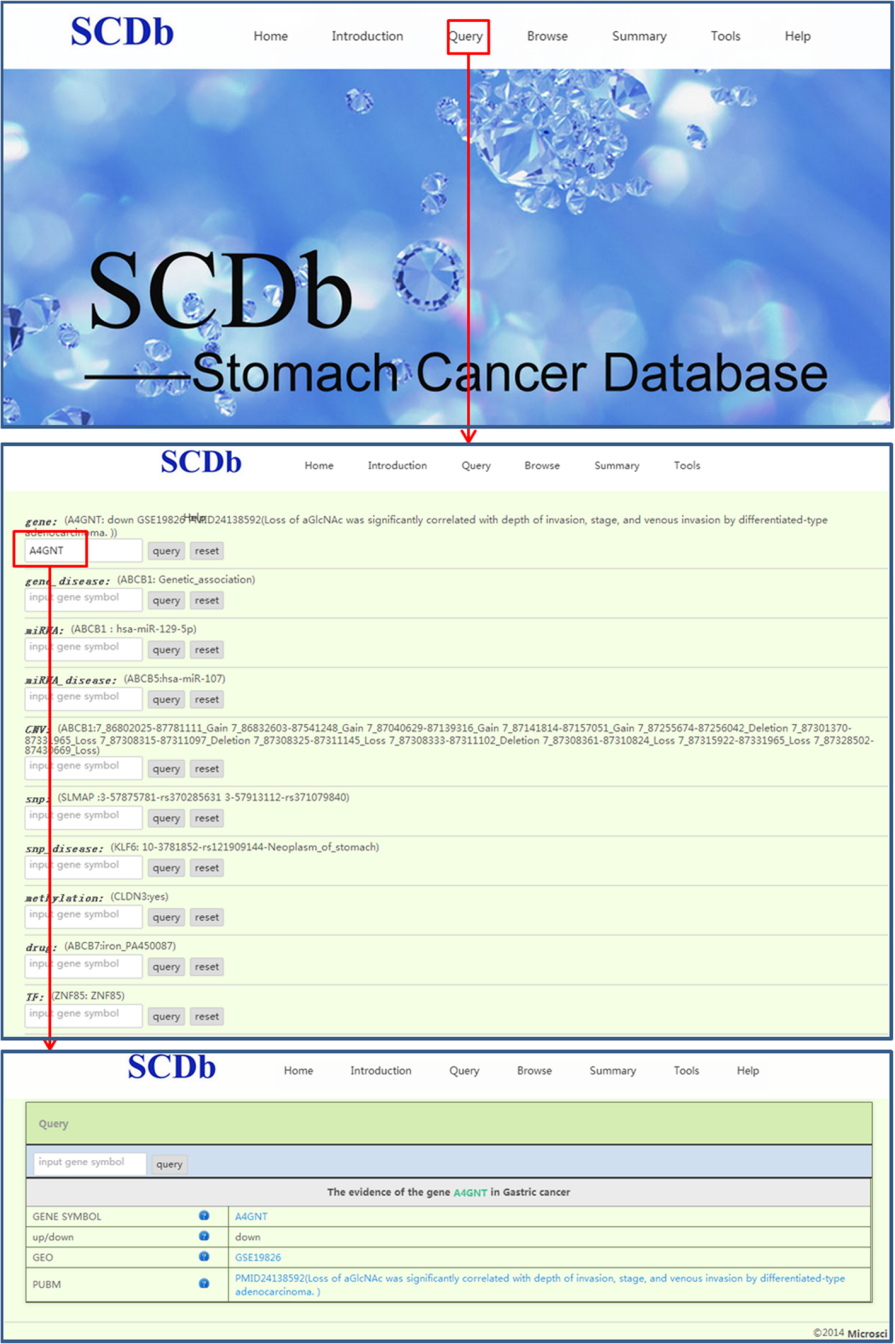
The flowchart of Query page. SCDb, Database of Stomach Cancer; GEO, Gene Expression Omnibus; SNP, single nucleotide polymorphism; TF, transcription factor; CNV, copy number variation; miRNA, microRNA

On the “Query” page, the search boxes for gene, gene_disease, miRNA, miRNA_disease, CNV, SNP, SNP_disease, methylation, drug and TF are listed from top to bottom as a drop down menu. After providing the input for gene symbol and clicking on query, information related to the sample content in the parentheses will be displayed on a new page. Further clicking on the terms in blue will link to the new pages in NCBI Gene, NCBI PubMed or NCBI GEO databases, which further describes the corresponding terms in detail. The flowchart of the usage of “Query” is presented in Fig. 2.

**Fig. 3:**
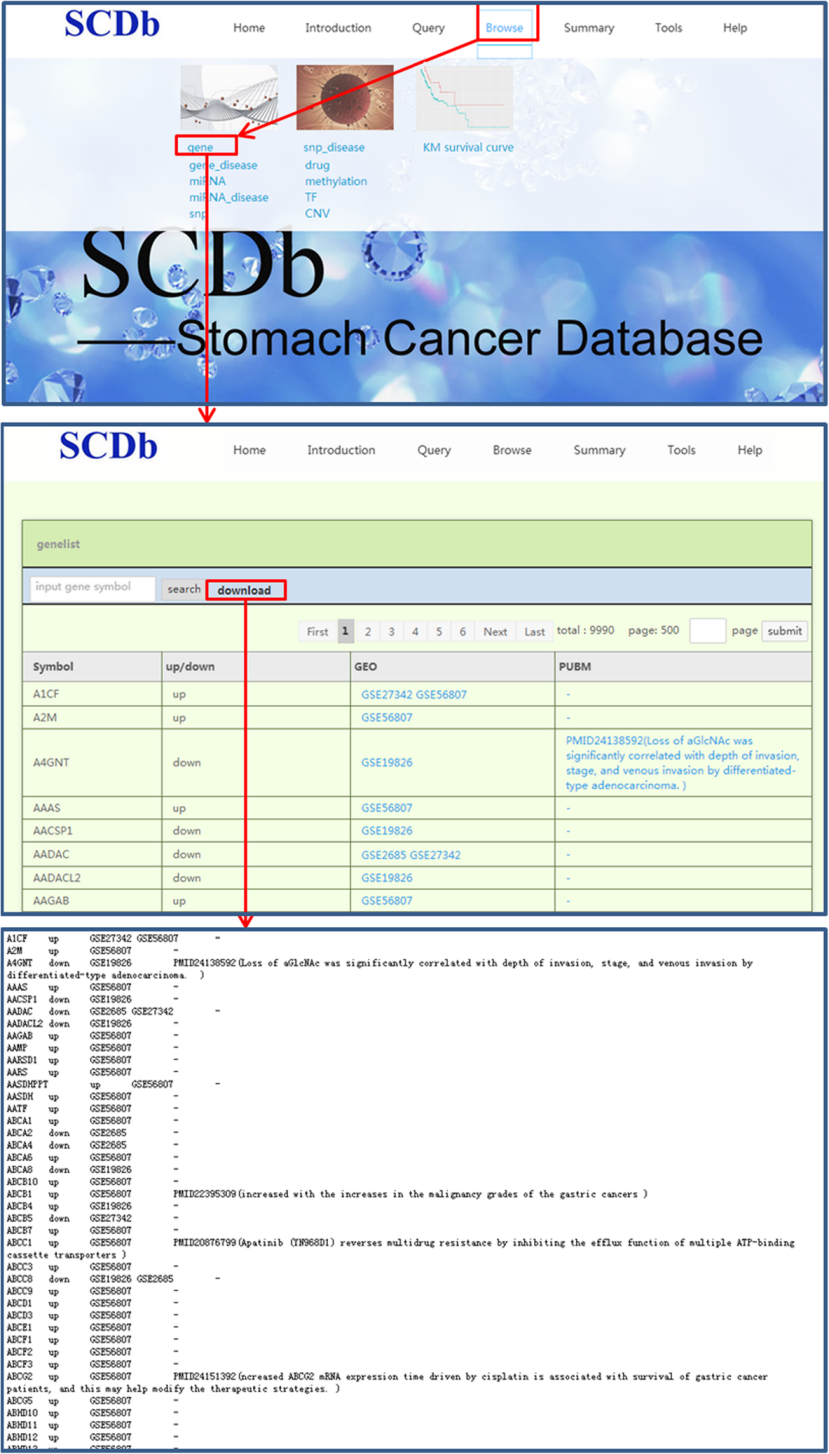
The flowchart of Browse page. SCDb, Database of Stomach Cancer; GEO, Gene Expression Omnibus; SNP, single nucleotide polymorphism; TF, transcription factor; CNV, copy number variation; miRNA, microRNA; KM: Kaplan-Meier

The “Browse” page also includes the terms gene, gene_disease, miRNA, miRNA_disease, CNV, SNP, SNP_disease, methylation, drug, TF, and KM survival curves. The usage of “Browse” page is very similar to that of “Query” page, except that all of the corresponding information for each term included in the SCDb database will appear just by clicking on the download button appearing after the search box. The flowchart of the usage of “Browse” is presented in Fig. 3.

**Fig. 4:**
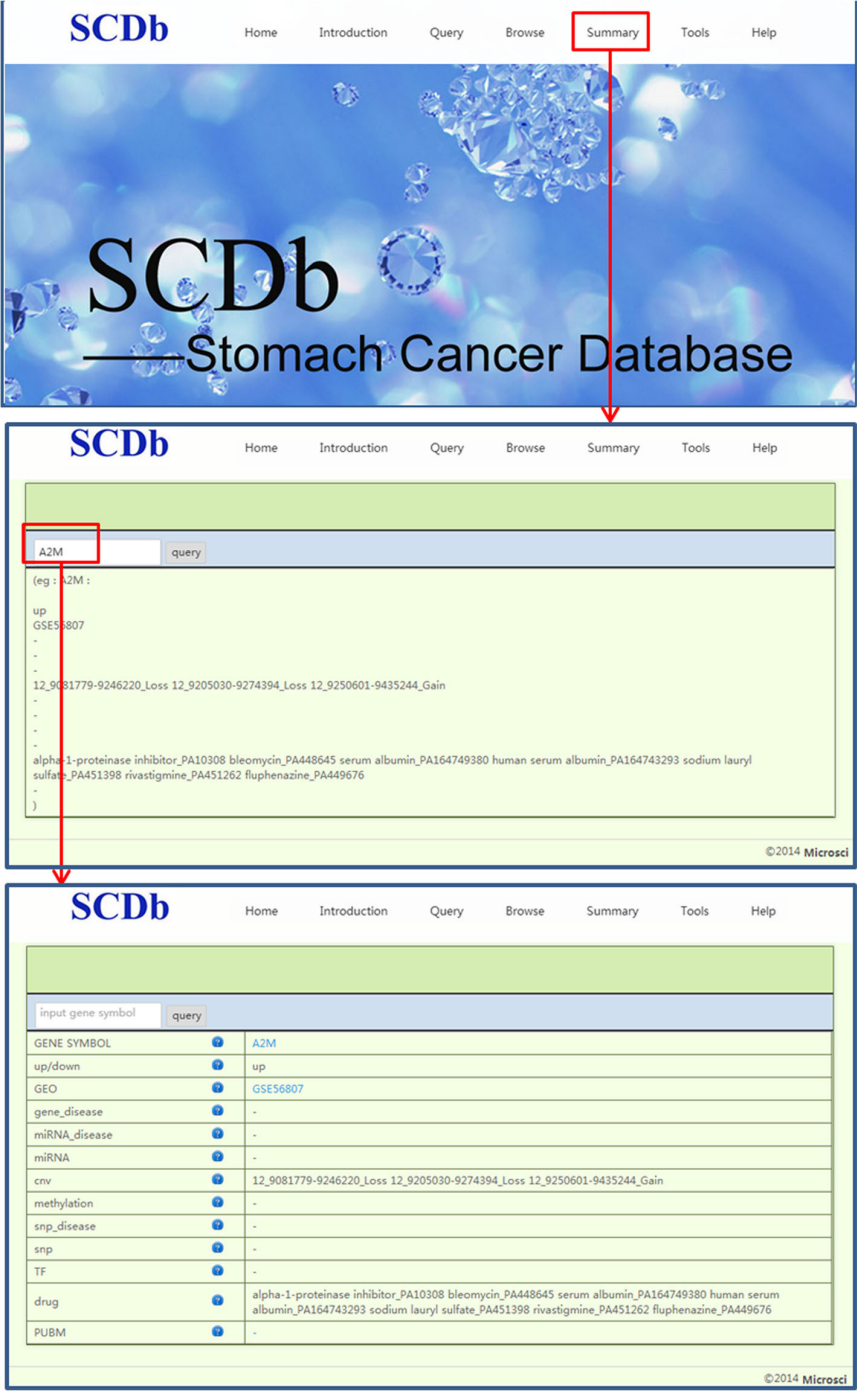
The flowchart of Summary page. SCDb, Database of Stomach Cancer; GEO, Gene Expression Omnibus; SNP, single nucleotide polymorphism; TF, transcription factor; CNV, copy number variation; miRNA, microRNA

On the “Summary” page, upon providing the input of a gene symbol and clicking on the query button, one can find all the information related to the gene, including its up−/down-regulation status, GEO, gene_disease, miRNA_disease, miRNA, CNV, methylation, SNP_disease, SNP, TF, drug and PUBMterms. The flowchart of the usage of “Summary” is presented in Fig. 4.

**Fig. 5:**
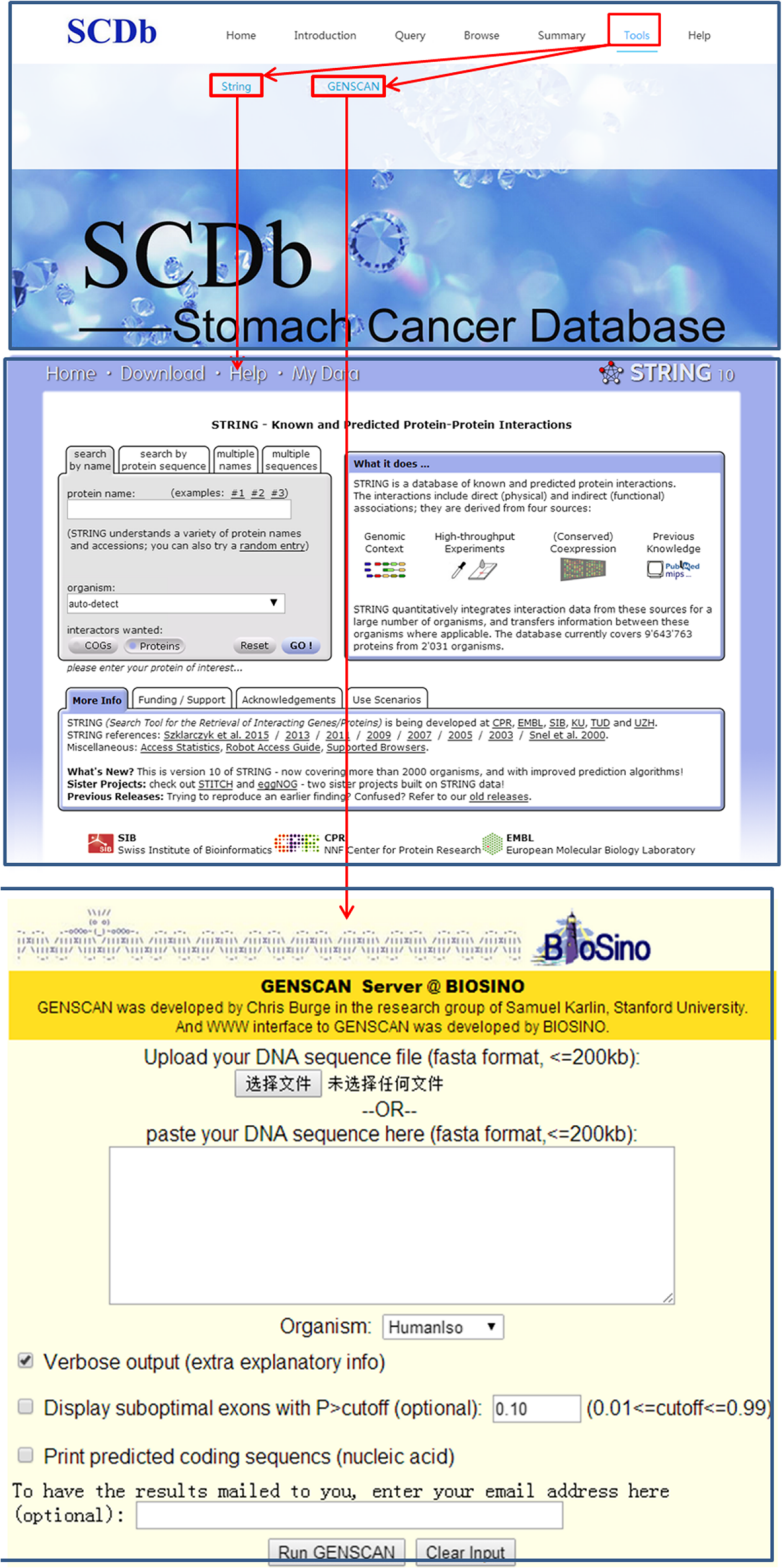
The flowchart of Tools page

On the “Tools” page, String (http://www.string-db.org) and GENSCAN (http://hollywood.mit.edu/GENSCAN.html) terms are included. After clicking on the terms, a new page of String or GENSCAN will appear directly. The flowchart of the usage of “Tools” is presented in Fig. 5.

## Discussion

As there are non-specific symptoms or no such noticeable symptoms observed in early stages of SC, newly diagnosed SC cases usually reach an advanced stage and are thus difficult to cure. To better understand the pathogenesis of SC, we developed the SCDb database that includes information on SC-related genes, gene_disease, miRNA, miRNA_disease, CNV, SNP, SNP_disease, methylation, drug, TF and KM survival curves. All this information was retrieved by analyzing the microarray datasets and by mining the literature. Information on SC-related genes (eg. gene symbol, up−/down- regulation, GEO ID and PUBM ID), gene_disease (eg. gene symbol and gene_disease), miRNA (eg. gene symbol and miRNA symbol), miRNA_disease (eg. gene symbol and miRNA symbol), CNV (eg. gene symbol and CNV), SNP (eg. gene symbol and SNP), SNP_disease (eg. gene symbol and SNP_disease), methylation (eg. gene symbol and methylation), drug (eg. gene symbol and drug), and TF (eg. gene symbol and TF) were integrated into this database. At present, the database includes information of 9990 SC-related genes, 65 confirmed SC-related genes, 457 miRNAs, 20 SC-related miRNAs, 1570 SNPs, 108 SC-related SNPs, 419 TFs, 44,605 CNVs, 3404 drug-associated genes, 63 genes with methylation and KM survival curves of 20,264 genes.

Compared to the previously established DBGC database [16], the SCDb database has several advantages: (1) SCDb database includes not just previously established information i.e. specifically, by performing the analyses using the microarray datasets, and RNA-seq datasets, novel genes, miRNAs, and SNPs were identified, which can further contribute to the determination of new directions for SC-related research; (2) SCDb database provides detailed regulatory information, for instance, possible TF-gene and miRNA-gene pairs associated with SC might also be identified based on the SC-related genes information; (3) a comprehensive analysis was performed for the SC-related genes, and various other data were integrated into the database, including the information on gene, gene_disease, miRNA, miRNA_disease, CNV, SNP, SNP_disease, methylation, drug, TF, and KM survival curves; (4) SCDb provides a search engine for tools, including String and GENSCAN, and thus protein-protein interaction analysis and gene prediction for unknown sequences can also be performed using SCDb; (5) SCDb provides search engines for Query, Browse, and Summary. Therefore, we can not only perform a search for gene, gene_disease, miRNA, miRNA_disease, CNV, SNP, SNP_disease, methylation, drug, TF terms, and KM survival curves in detail but can also obtain all the corresponding information of each term that is included in the SCDb database and all information related to one gene.

However, the gene expression data that were collected from multiple publicly available microarray datasets and more details of these datasets, such as number of patients, ethnicity of patients, and how the samples were prepared were not provided, which might be potential limiting factors influencing our results. Moreover, with the advancements in sequencing techniques, the microarray data about SC might not be constantly updated in GEO, and therefore, next-generation data about SC should be obtained, which might provide new insights into SC biology, and should be added if available. Furthermore, this established SCDb database does not provide any information on gene expression based on clinical parameters, such as age, gender, histological or molecular subtypes, tumor stage or grading, and prior therapies. Lastly, we did not conduct the analysis of the correlation between cancer progression stages with gene expression data as well as the multivariate analysis to detect more specific prognostic markers for survival. Considering these limitations, we plan to update the database periodically to continuously improve the quality of the SC-related data and the corresponding functions, thus keeping a track of improvements and advancements in this field.

## Conclusion

In conclusion, the SCDb database provides a comprehensive resource for performing research on human SC. We believe that SCDb will be a helpful database for biologists and pharmacologists in the field of SC research, and will promote the studies to better understand the molecular mechanisms of this disease.

## Supplementary information

**Additional file 1.**

## Data Availability

The datasets used and analyzed in the current study are available from the corresponding author in response to reasonable requests.

## Abbreviations

CNVs: Copy number variations
DBGC: Database of Human Gastric Cancer
DEGs: Differentially expressed genes
DGV: Database of Genomic Variants
FC: Fold change
GAD: Genetic association database
GEO: Gene Expression Omnibus
KM: Kaplan-Meier
NCBI: National Center for Biotechnology Information
OMIM: Online Mendelian Inheritance in Man
SC: Stomach cancer
SNP: Single nucleotide polymorphism
TCGA: The Cancer Genome Atlas
TF: Transcription factor
